# Jianpi Qingchang Decoction Ameliorates Chronic Colitis in Piroxicam-Induced IL-10 Knockout Mice by Inhibiting Endoplasmic Reticulum Stress

**DOI:** 10.1155/2022/7378807

**Published:** 2022-02-09

**Authors:** Qian Chen, Ya-Li Zhang, Zi-Wei Zhang, Yu-Jun Chen, Ying-Jue Tang, Dan Qiao, Yan-Cheng Dai, Zhi-Peng Tang

**Affiliations:** ^1^Institute of Digestive Disease, Longhua Hospital, Shanghai University of Traditional Chinese Medicine, Shanghai 200032, China; ^2^Department of Gastroenterology, Shanghai Traditional Chinese Medicine Integrated Hospital, Shanghai University of Traditional Chinese Medicine, Shanghai 200082, China

## Abstract

**Background:**

Excessive endoplasmic reticulum (ER) stress in intestinal epithelial cells (IEC) may lead to impaired intestinal mucosal barrier function and then participate in the pathogenesis of ulcerative colitis (UC). Jianpi Qingchang decoction (JPQCD) has been shown to have protective effects on UC. However, further studies are needed to determine whether JPQCD regulates PERK/eIF2*α*/ATF4/CHOP pathways to play a role in treating UC.

**Methods:**

*IL-10* ^−/−^ mice were randomly assigned into five groups: control, model, low-dose JPQCD (JPQCD L), middle-dose JPQCD (JPQCD M), and high-dose JPQCD (JPQCD H). All groups except for the control group were given model feed containing 200 ppm piroxicam for 10 d to induce colitis. As a comparison, we used wild-type mice that were the progeny of *IL-10*^+/−^ matings, bred in the same facility. The control group and wild-type mice were fed with common feed. At the same time, mice in each group were given corresponding drugs by gavage for 14 d. The disease activity index of mice in each group was evaluated daily. Colon tissues of mice were collected, colon length was measured, and pathological changes and ultrastructure of colon epithelial cells were observed. The effects of JPQCD on the PERK/eIF2*α*/ATF4/CHOP pathways were evaluated by western blotting and reverse transcription-polymerase chain reaction (RT-PCR). The expression of CHOP in colon tissue was detected by tissue immunofluorescence assay. The expression of NF-*κ*B, p-NF-*κ*B p65 protein was analyzed by western blotting; the level of IL-17 in colon tissue was detected by enzyme-linked immunosorbent assay (ELISA) and verified by examining NF-*κ*B and IL-17 mRNA levels by RT-PCR.

**Results:**

Compared with the control group, the model group showed significant colitis symptoms and severe colonic tissue damage. The results showed that JPQCD significantly reduced body weight loss, ameliorated disease activity index, and restored colon length in *IL-10*^−/−^ mice with piroxicam-induced colitis. Western blotting and RT-PCR showed that the PERK/eIF2*α*/ATF4/CHOP pathway was activated in colon tissue of model mice, suggesting that the pathway is involved in the pathogenesis of ulcerative colitis (UC) and could become a potential therapeutic target. The JPQCD treatment inhibited the activation of the PERK/eIF2*α*/ATF4/CHOP pathway, alleviated the ER stress, and played a role in preventing and treating UC. In addition, JPQCD can also downregulate the protein of NF-*κ*B, p-NF-*κ*B p65, downregulate the mRNA expression of NF-*κ*B, and reduce the content of IL-17 and its mRNA expression in colon tissues.

**Conclusion:**

JPQCD may play a protective role in UC by regulating the PERK/eIF2*α*/ATF4/CHOP signaling pathway and relieving endoplasmic reticulum stress.

## 1. Introduction

Ulcerative colitis (UC) is a chronic nonspecific intestinal inflammatory disease characterized by continuous and diffuse inflammatory changes in the colorectal mucosa. Its lesions are mainly confined to the large intestinal mucosa and submucosa. Clinical manifestations are diarrhea, mucous pus and blood in the stools, and abdominal pain. The severity of the disease varies, and most cases show a chronic course of recurrent attacks [1]. The incidence and prevalence of UC are increasing worldwide. The etiology of UC is not clear, and it is generally considered to be related to factors such as heredity, environment, intestinal microecology, and immune imbalance [2]. So far, aminosalicylic acid preparations, corticosteroids, and immunosuppressants are the main drugs for the treatment of UC. However, these drugs are accompanied by a variety of potential adverse effects [3]. As an alternative and complementary medicine for the treatment of inflammatory bowel disease (IBD), traditional Chinese medicine (TCM) has unique advantages for the prevention and treatment of IBD due to its efficacy and safety [4, 5].

It has been shown that various causes contribute to intestinal epithelial cell (IEC) endoplasmic reticulum (ER) stress and activate the unfolded protein response (UPR), which collectively participate in the development of IBD [6, 7]. UPR is controlled by three major sensors: pancreatic ER eIF2*α* kinase (PERK), inositol-requiring enzyme 1 (IRE1), and activating transcription factor 6 (ATF6) [8]. In the absence of ER stress, glucose-regulated protein 78 (GRP78) binds to the luminal domains of the ER stress sensors and is maintained in an inactive state [9]. Upon ER stress, GRP78 dissociates from three proteins, thus activating PERK, IRE1, or ATF6 and starting UPR, thus promoting the correct folding of proteins and inhibiting the synthesis of proteins [10]. Among the three pathways of ER stress, the PERK pathway mainly recognizes and interacts with a variety of unfolded proteins to regulate protein synthesis [11].

Jianpi Qingchang decoction (JPQCD) is composed of *Coptis chinensis* Franch. (Huang Lian) 3 g, *Astragalus mongholicus Bunge* (Huang Qi) 30 g, *Codonopsis pilosula* (Franch.) Nannf. (Dang Shen) 15 g, *Portulaca oleracea* L. (Ma Chi Xian) 30 g, *Sanguisorba officinalis* L. (Sheng Di Yu) 15 g, *Panax notoginseng* (Burkill) F. H. Chen (San Qi) 6 g, *Bletilla striata* (Thunb.) Rchb.f. (Bai Ji) 3 g, *Aucklandia costus* Falc. (Mu Xiang) 6 g, and *Glycyrrhiza uralensis* Fisch. *ex* DC. (Gan Cao) 6 g. Previous studies have shown that JPQCD can significantly alleviate the clinical symptoms of patients with mild-to-moderate active UC and thus improve their quality of life [12]. Previous experimental studies have shown that JPQCD can significantly improve the symptoms of dextran sodium sulfate- (DSS-) induced colitis, and the mechanism may be related to the inhibition of nuclear factor- (NF-) *κ*B activation, downregulation of inflammatory mediators such as interleukin- (IL-) 1*β*, IL-8, tumor necrosis factor-*α*, and improvement of colonic epithelial barrier function in mice [13, 14]. Moreover, we found that JPQCD can also regulate DSS-induced abnormal intestinal motility in UC mice by inhibiting intestinal inflammatory cascading and reducing autophagy of Cajal stromal cells [15]. However, it is not clear whether JPQCD can regulate the PERK/eIF2*α*/ATF4/CHOP pathway. In this study, we used piroxicam to induce *IL-10*^−/−^ mice to produce a chronic colitis model and explored whether JPQCD could regulate the PERK/eIF2*α*/ATF4/CHOP pathway, thus improving ER stress and preventing UC.

## 2. Materials and Methods

### 2.1. Animals and Experimental Design

*IL-10* ^−/−^ mice on a C57BL/6 strain background were obtained from Shanghai Model Organisms (license No. SCXK (Shanghai) 2017-0010). Mice were bred and mated in SPF Animal Experimental Center of Shanghai University of Traditional Chinese Medicine (license No. SCXK (Shanghai) 2020-0009), under standard conditions (room temperature, 24 ± 2°C; humidity 50%–60%; 12 h light/dark cycle). Shanghai Model Organisms provided genetic testing services. This study was reviewed and approved by the Experimental Animal Ethics Committee of Shanghai University of Chinese Medicine (PZSHUTCM190912020).

Using SPSS version 25.0 to generate random numbers, *IL-10*^−/−^ mice were randomly assigned to five groups: control, model, low-dose JPQCD (JPQCD L), middle-dose JPQCD (JPQCD M), and high-dose JPQCD (JPQCD H) (*n* = 8, 4 male and 4 female). Chow containing piroxicam (Sigma–Aldrich, St. Louis, MO, USA) was fed to *IL-10*^−/−^ mice for 10 d at a dose of 200 ppm to induce colitis [16]. For comparison, we used wild-type mice that were the progeny of *IL-10*^+/−^ matings, bred in the same facility. Control and wild-type mice were fed a common diet. Mice in the treatment group were given JPQCD (10, 15, or 22.5 g/kg/d) by gavage for 2 wk, and mice in the model group, control group, and wild-type group were given normal saline by gavage. The animal experiment was conducted in accordance with the Laboratory Animal Regulations of the Institutional Animal Care and Use Committee of Shanghai University of Traditional Chinese Medicine and was approved by the Institutional Animal Care and Use Committee of Shanghai University of Traditional Chinese Medicine. During the experiment, the body weight, stool characteristics, and fecal occult blood of mice in each group were recorded every day. The disease activity index (DAI) refers to Sánchez's method and is described in Table 1, which is composed of the mean value of the sum of the three indexes [17].

### 2.2. Preparation of JPQCD

The nine medicinal herbs contained in JPQCD (listed above) were purchased from Longhua Hospital Shanghai University of Traditional Chinese Medicine (Shanghai, China). After soaking the above medicines, they were decocted twice, filtered, and concentrated to a volume of 600 mL. After being concentrated in a rotary evaporator, the concentrated JPQCD was placed in a freeze-drying machine to prepare freeze-dried powder, which was sealed and stored at −20°C.

### 2.3. Ultraperformance Liquid Chromatography Quadrupole-Time of Flight Mass Spectrometer (UPLC-Q-TOF/MS)

Accurately weigh 0.5 g of JPQCD powder into a 50 mL stoppered conical flask and add 25 ml of 80% methanol solution, ultrasonicated for 30 min, cooled to room temperature, centrifuged at 12000 rpm for 5 min, and the supernatant was taken for detection.

Chemical profiling of JPQCD was performed on an Agilent 1290 UPLC System (Agilent Technologies, Palo Alto, USA) coupled with Sciex Triple TOF® 4600 high-resolution mass spectrum (AB Sciex, Darmstadt, Germany). Chromatographic column using Agilent ZORBAX RRHD Eclipse XDB-C18 (2.1 × 100 mm, 1.8 *µ*m). The mobile phase consisted of water containing 0.1% formic acid (A) and acetonitrile (B). The following gradient condition was used: 0–5 min, 5%–5% B; 5–7 min, 5%–10% B; 7–23 min 10%–15% B; 23–36 min, 15%–30% B; 36–48 min, 30%–50% B; 48-50 min, 50%–95% B; 50-52 min, 95%–95% B; 52–52.1 min, 95%–5% B; 52.1–54 min, 5%–5% B. Column oven temperature was set at 30°C, while the flow rate was 0.3 mL/min. Mass spectrometry detection mode is electrospray ionization (ESI) source negative/positive ion mode.

### 2.4. Histological Analysis

Colon tissue from the anus to the ileocecal part of mice was cut and colon length was measured with a ruler. The distal colon was collected 1 cm from the anus and about 0.5 cm in length for histopathological analysis. Colon tissue was fixed with 10% neutral formalin buffer solution, washed for 4 h under running water, embedded in paraffin, and stained with hematoxylin and eosin (H&E). The specific steps were as follows: dehydration with graded ethanol, Xylene, and wax immersion, embedding in paraffin; cutting into 4 *μ*m sections, baking at 60°C for 1 h, H&E staining, sealing with neutral gum, observing under a microscope, and taking pictures.

### 2.5. Transmission Electron Microscopy

The fresh colon tissue was cut into 1-mm^3^ pieces, fixed in 2.5% glutaraldehyde for 2 h, and fixed in 1% osmium at 4°C for 3 h. The tissue was dehydrated with graded ethanol and acetone, and embedded and cured. Ultrathin sections were cut with a thickness of 50–60 nm, and double-stained with 3% uranyl acetate and lead citrate. The ultrastructure of the ER in colonic epithelial cells was observed by transmission electron microscopy.

### 2.6. RNA Isolation and Quantitative Reverse Transcription-Polymerase Chain Reaction (RT-PCR)

Total RNA was extracted from colon tissue using TRIzol (Invitrogen, Carlsbad, CA, USA) and the concentration was determined. The extracted RNA was reverse transcribed to cDNA using a reverse transcription kit (Takara, Kyoto, Japan) and RT-PCR was conducted based on the Eppendorf PCR system. The mRNA expression of the following genes was assessed using qPCR: *β-actin*, *PERK*, *eIF2α*, *GRP78*, *ATF4*, *CHOP, NF-κB, IL-17*. The relative expression of target genes was calculated using the △△Ct method. Primer sequences are presented in Table 2.

### 2.7. Western Blotting

Frozen colon tissue samples were homogenized in lysis buffer to obtain total protein. Protein concentration was determined in supernatants using the BCA Protein concentration determination kit (Beyotime, Shanghai, China). Equal amounts of protein were separated by SDS-PAGE and transferred to the polyvinylidene difluoride membrane. The membranes were blocked in 5% nonfat milk for 1 h, and membranes were incubated at 4°C overnight with primary antibodies to p-PERK, eIF2*α*, p-eIF2*α*, CHOP, NF-*κ*B, *β*-actin (from Cell Signaling, Danvers, MA, USA), GRP78 (from Proteintech, Wuhan, Hubei, China), ATF4 (from Santa Cruz, Dallas, TX, USA), and p-NF-*κ*B p65 (from ABclonal, Wuhan, Hubei, China). The membranes were incubated with secondary antibodies, anti-rabbit IgG or anti-mouse IgG (from Cell Signaling, Danvers, MA, USA) for 1 h at room temperature. Protein bands were quantified using ImageJ and the grey value of the targets was normalized by *β*-actin.

### 2.8. Immunofluorescence

To investigate localization of CHOP, paraffin sections of the colon were dewaxed to water, antigen repaired, blocked, and then incubated with CHOP (1 : 400) overnight. The secondary antibody was incubated at room temperature for 1 h and then stained with DAPI for 2 min.

### 2.9. Enzyme-Linked Immunosorbent Assay

The colon tissue of 50 mg mice was weighed and added 1 mL of precooled PBS. After homogenization, the supernatant was centrifuged at 5000x g for 5 min. Experiments were done following the instructions of the Mouse IL-17 ELISA kit (from Elabscience, Wuhan, Hubei, China).

### 2.10. Statistical Analysis

All data are presented as mean ± SEM. Statistical differences between different groups were measured through one-way ANOVA. Results were considered statistically significant at *P* < 0.05. Data analyses were conducted using SPSS version 25.0.

## 3. Results

### 3.1. Phytochemicals Identification of JPQCD

**Figure 1:**
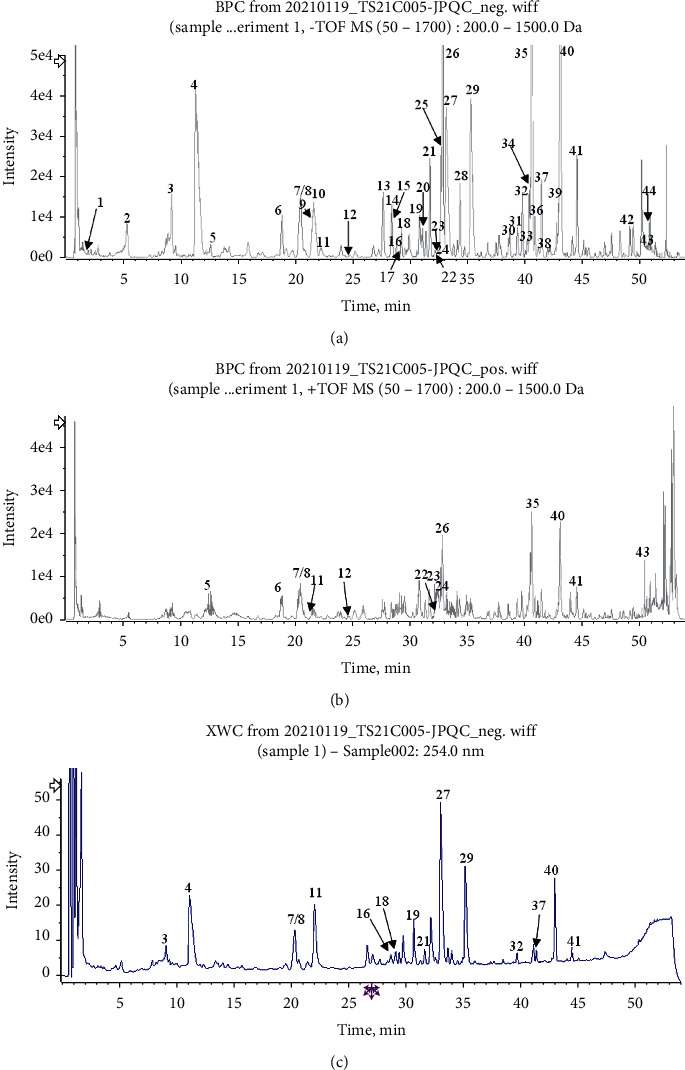
UPLC-Q-TOF/MS of JPQCD. (a) UPLC-HRMS base peak ion flow graph (BPC) negative ion mode for JPQCD; (b) UPLC-HRMS BPC-positive ion mode for JPQCD; (c) UPLC UV xhromatogram of JPQCD-UV 254 nm.

Forty-four phytochemicals in JPQCD were identified by UPLC-Q-TOF/MS-based on multistage mass spectrum information of samples, high-resolution mass spectrum database of natural products, and relevant literature (Table 3). Among them, 9 compounds were from *Glycyrrhiza uralensis* Fisch. *ex* DC., 8 compounds from *Sanguisorba officinalis* L., and 8 compounds from *Panax notoginseng* (Burkill) F. H. Chen. Five compounds are derived from *Bletilla striata (*Thunb.) Rchb.f.. Then, there were 4, 3, 3, 2, and 1 compounds derived from *Coptis chinensis* Franch., *Portulaca oleracea* L., *Astragalus mongholicus* Bunge, *Aucklandia costus* Falc., and *Codonopsis pilosula* (Franch.) Nannf. The UPLC-Q-TOF/MS chromatographic profile is shown in Figure 1.

### 3.2. JPQCD Alleviated the Symptoms of Experimental Chronic Colitis in IL-10^−/−^ Mice

**Figure 2:**
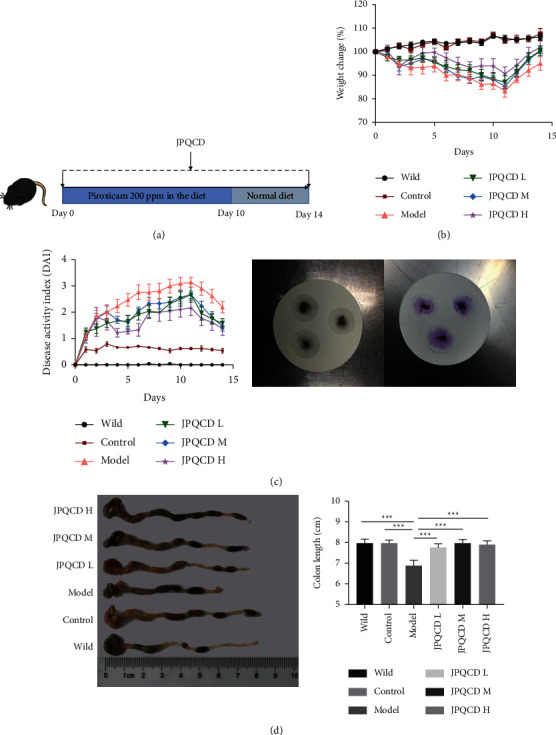
JPQCD can improve the symptoms of experimental chronic colitis in *IL-10*^−/−^ mice: (a) animal experiment design. *IL-10*^−/−^ mice were induced by piroxicam for 10 days. Wild-type/control group/model group and JPQCD group were given normal saline or JPQCD daily. *n* = 6–8. (b) The body weight was measured every day. (c) Schematic diagram of disease activity index score and fecal occult blood test. (d) Colon length and statistics. Data are shown as the mean ± SEM. Compared to the model group, ^*∗*^*P* < 0.05, ^*∗∗*^*P* < 0.01, ^*∗∗∗*^*P* < 0.001.

We established a mouse model of chronic colitis by adding 200 ppm piroxicam feed to *IL-10*^−/−^ mice for 10 d to evaluate the therapeutic effect of JPQCD (Figure 2(a)). *IL-10*^−/−^ mice exposed to piroxicam showed significant weight loss, diarrhea, and blood in the stools. DAI of mice in the model group was higher than that in the wild-type group and control group, and JPQCD treatment significantly increased body weight and reduced DAI (Figures 2(b) and 2(c)). Colonic shortening indirectly reflected the pathological process of colitis, and JPQCD significantly alleviated piroxicam-induced colonic shortening in mice (Figure 2(d)).

### 3.3. JPQCD Inhibited Intestinal Inflammatory Infiltration of Experimental Chronic Colitis in IL-10^−/−^ Mice

**Figure 3:**
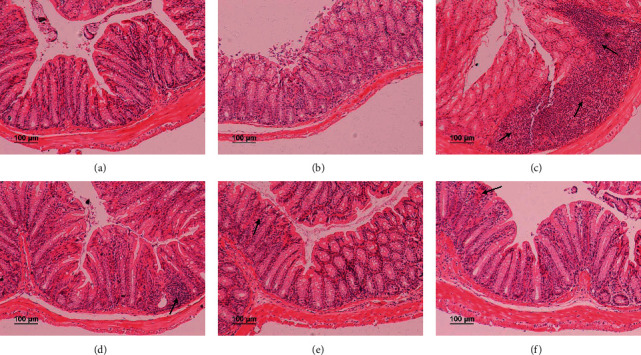
Histologic images of mice colon (H&E, magnification ×100). (a) Wild-type group; (b) the control group; (c) model group; (d) low-dose JPQCD group; (e) middle-dose JPQCD group; (f) high-dose JPQCD group. The arrow represents the infiltration of inflammatory cells.

H&E staining showed that the colon crypts of wild-type mice were normal, with abundant goblet cells, few lamina propria monocytes, and no sign of mucosal thickening or ulceration. In the model group, there was a lot of inflammatory cell infiltration, mucosal thickening, goblet cell reduction, obvious ulceration, and irregular glandular arrangement in the colonic tissues. The JPQCD-treated mice showed intact colonic structures, no obvious ulcers, and less inflammatory cell infiltration (Figures 3(a)–3(f)).

### 3.4. JPQCD Reduced ER Stress in IECs of Mice with Piroxicam-Induced Colitis

**Figure 4:**
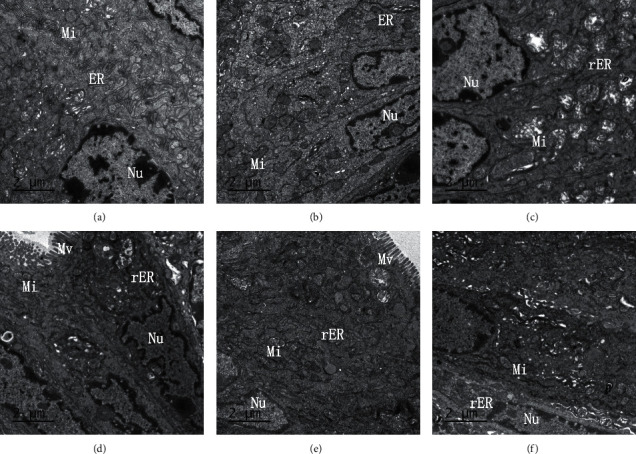
Transmission electron microscopy of mouse intestinal epithelial cells (magnification ×6000). (a) Wild-type group; (b) the control group; (c) model group; (d) low-dose JPQCD group; (e) middle-dose JPQCD group; (f) high-dose JPQCD group. Nu: nucleus; Mi: mitochondrial; ER: endoplasmic reticulum; rER: rough endoplasmic reticulum; Mv: microvillus.

The colonic epithelial cells were observed under transmission electron microscopy. In the wild-type and control groups, the ER showed a membranous network structure, clear and in clumps; the omental cavity was not expanded, and a large number of ribosomes were attached to it; and the cells had many mitochondria with a light color. In the model group, the rough ER (RER) was increased, and the ER cavity was significantly expanded. There were many ER cavities of different sizes and shapes, which were vacuolated and partially fused into clusters. In the JPQCD group, the RER was reduced compared with that in the model group and slightly dilated with less quantity, and the morphology tended to be reticular. In conclusion, JPQCD improved ER stress in IECs of mice with colitis (Figures 4(a)–4(f)).

### 3.5. JPQCD Regulated Gene Expression Related to PERK/eIF2*α*/ATF4/CHOP Signaling Pathway

**Figure 5:**
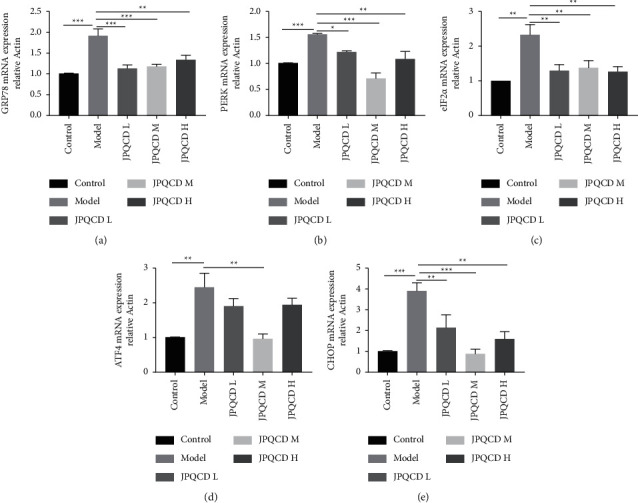
Effects of Jianpi Qingchang decoction (JPQCD) on the expression of GRP78, PERK, eIF2*α*, ATF4, and CHOP mRNA by RT-PCR in piroxicam-induced colitis *IL-10*^−/−^ mice. Data are shown as the mean ± SEM. Compared to the model group, ^*∗*^*P* < 0.05, ^*∗∗*^*P* < 0.01, ^*∗∗∗*^*P* < 0.001.

To further investigate the effect of JPQCD on piroxicam-induced colitis, we performed an RT-PCR analysis of several signaling molecules. Compared with the control group, the mRNA levels of PERK, eIF2*α*, GRP78, ATF4, and CHOP in the model group were higher. The mRNA expression of PERK, eIF2*α*, GRP78, ATF4, and CHOP in the colon of the JPQCD group was significantly lower than that in the model group. These results suggested that JPQCD-induced improvement in experimental colitis in mice was associated with the PERK/eIF2*α*/ATF4/CHOP signaling pathway (Figures 5(a)–5(f)).

### 3.6. JPQCD Regulated Expression of Proteins Related to the PERK/eIF2*α*/ATF4/CHOP Signaling Pathway

**Figure 6:**
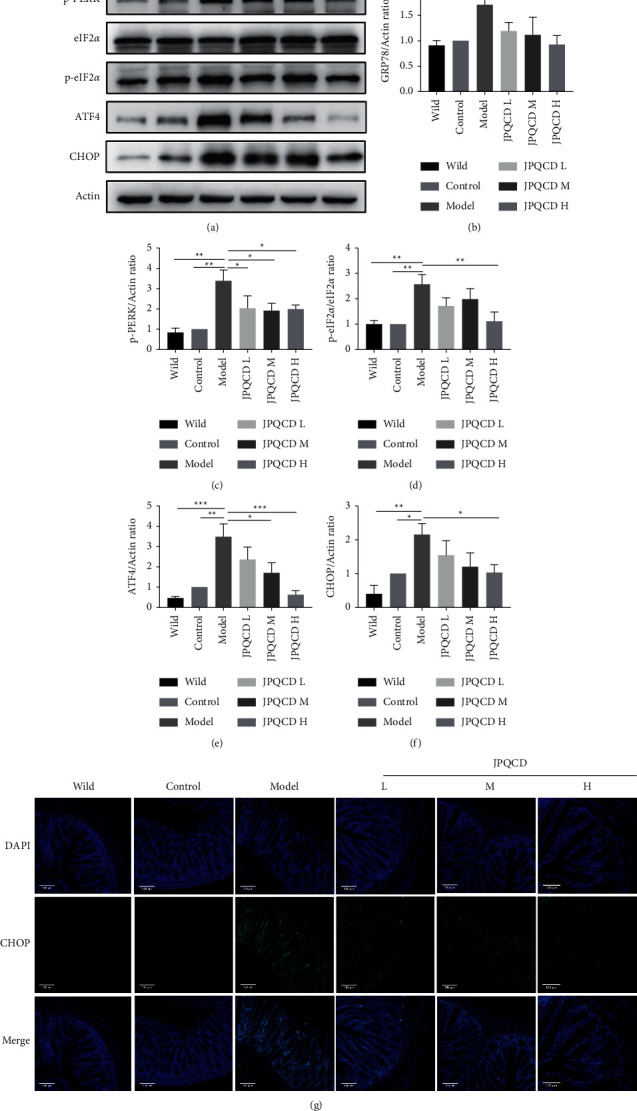
(a) Effects of Jianpi Qingchang decoction (JPQCD) on the expression of PERK/eIF2*α*/ATF4/CHOP pathway proteins assessed by western blot in piroxicam-induced colitis *IL-10*^−/−^ mice. (b-f) Densitometric analysis was performed to determine each protein. *β*-Actin was used as the loading control. Data are shown as the mean ± SEM. Compared to the model group, ^*∗*^*P* < 0.05, ^*∗∗*^*P* < 0.01, ^*∗∗∗*^*P* < 0.001. (g) Immunofluorescence staining for CHOP in colon tissues (magnification ×100).

To further investigate the anti-inflammatory mechanism of JPQCD, we detected the protein expression of key signaling molecules in the PERK/eIF2*α*/ATF4/CHOP pathway. Western blotting showed that PERK, eIF2*α*, GRP78, ATF4, and CHOP proteins were highly expressed in the model group, while JPQCD treatment significantly reduced the expression of these proteins. The results showed that the improvement of experimental colitis symptoms in mice induced by JPQCD was associated with inhibition of the PERK/eIF2*α*/ATF4/CHOP signaling pathway (Figures 6(a)–6(f)). Immunofluorescence staining further confirmed that JPQCD could depress CHOP expression in piroxicam-induced colitis mice (Figure 6(g)).

### 3.7. JPQCD Inhibited the Activation of NF-*κ*B and IL-17

**Figure 7:**
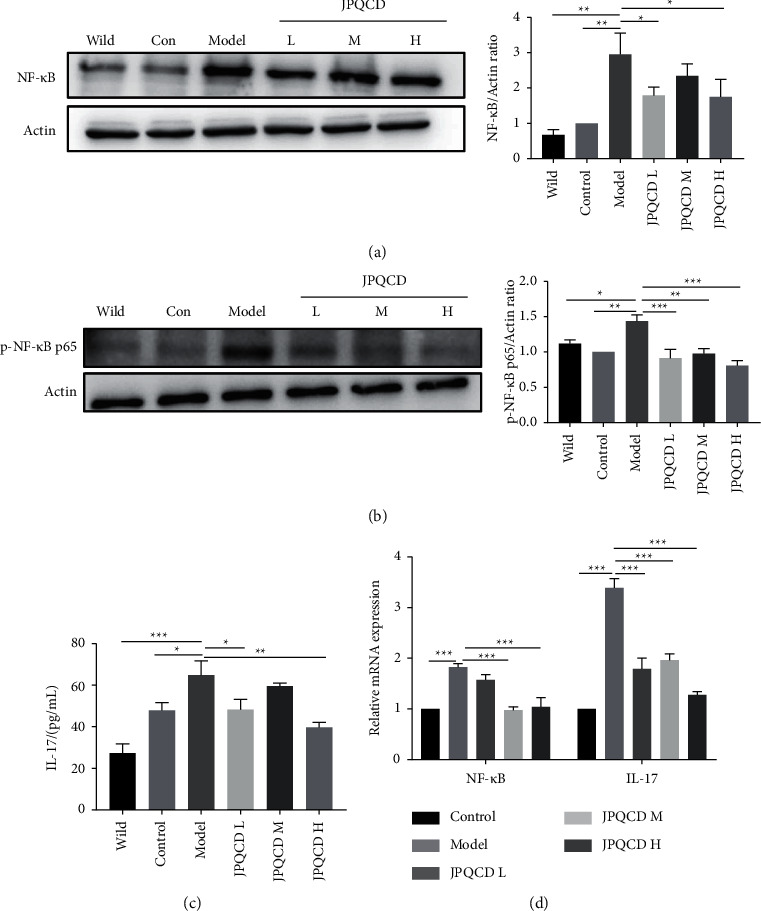
(a-b) Western blotting of NF-*κ*B, p-NF-*κ*B p65 in colon tissue. (c) Detection of IL-17 content in colon tissue by ELISA. (d) The mRNA levels of NF-*κ*B and IL-17 in colon tissue. Data are shown as the mean ± SEM. Compared to the model group, ^*∗*^*P* < 0.05, ^*∗∗*^*P* < 0.01, ^*∗∗∗*^*P* < 0.001.

**Figure 8:**
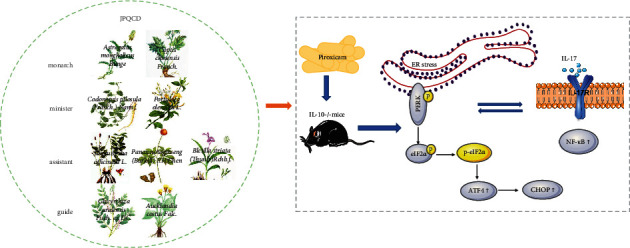
Schematic diagram of endoplasmic reticulum stress mechanism of Jianpi Qingchang decoction (JPQCD) in the process of improving piroxicam-induced chronic colitis in *IL-10*^−/−^ mice. (Plant illustrations originate from the Internet.)

NF-*κ*B is considered to be the key molecular pathway of UC. We detected the effects of JPQCD on the expression of NF-*κ*B by Western blot. The expression of NF-*κ*B protein increased significantly in the colon of piroxicam-induced colitis, which was inhibited by JPQCD treatment (Figure 7(a)). The protein level of p-NF-kB p65 reflected its transcription in the nucleus, and JPQCD significantly inhibited the phosphorylation level of p65 (Figure 7(b)). Since IL-17 is a cytokine with a strong proinflammatory activity involved in the pathogenesis of a variety of chronic inflammatory diseases, we used ELISA to detect the level of IL-17 in colonic tissues. A significant elevation of IL-17 content was observed in model mice compared with the control mice. The elevated IL-17 level was significantly decreased in colitis mice treated with JPQCD (Figure 7(c)). To further verify the inhibitory effects of JPQCD on NF-*κ*B and IL-17, we determined the mRNA levels of NF-*κ*B and IL-17 in the colon by RT-PCR. The results showed that the mRNA expressions of NF-*κ*B and IL-17 in the JPQCD group were significantly lower than those in the model group (Figure 7(d)). In summary, these data indicate that JPQCD can reduce the inflammatory response by alleviating endoplasmic reticulum stress and regulating the PERK/eIF2*α*/ATF4/CHOP signaling pathway, with the specific mechanism shown in Figure 8.

## 4. Discussion

UC is a chronic recurrent intestinal inflammatory disease classified as one of the refractory diseases by the World Health Organization. Due to the many adverse effects of current therapies, it is urgent to find an effective and safe treatment method for UC. The purpose of this study was to investigate the efficacy and potential mechanism of JPQCD in reducing piroxicam-induced chronic colitis in *IL-10*^−/−^ mice. We found that JPQCD repaired the intestinal mucosal barrier in mice, significantly reduced the piroxicam-induced colon inflammatory response, alleviated ER stress of colonic epithelial cells, and regulated the PERK/eIF2*α*/ATF4/CHOP pathway.

In our study, we used a chronic colitis model of *IL-10*^−/−^ mice induced by piroxicam. The *IL-10*^−/−^ mice is a genetic engineering model widely used for analyzing the causes of inflammatory bowel disease, which was established by Kühn et al. in 1993 [18]. In previous studies, we have demonstrated the efficacy of JPQCD in DSS-induced colitis in mice [13–15]. However, the use of chemical reagents to damage the colonic mucosal barrier of mice, increase its permeability, and then trigger the production of inflammatory lesions in the intestinal tract is significantly different from the mechanism of inflammatory bowel disease induced by diet, immunity, infection, spirit, and other factors in human UC, which limits the experimental research. The use of gene knockout technology to replicate animal models of UC has the characteristics of spontaneity and can simulate human UC, which is important for revealing the etiology, clarifying the genetic pathogenesis of the disease, and determining susceptibility genes [19]. It has been shown that, in C57BL/6 *IL-10*^−/−^ mice, spontaneous colitis development is slow, and the use of nonsteroidal anti-inflammatory drugs (NSAIDs) such as piroxicam can induce moderately severe colitis in 2 wk. Acute symptoms turned chronic after withdrawal of the drug, and its pathological features were consistent with those observed in the colonic tissues of spontaneous colitis mice, while wild-type mice exposed to NSAIDs did not develop colitis [20, 21]. The histopathological characteristics of *IL-10*^−/−^ mouse colitis are similar to those of human IBD, including lamellar and submucosal inflammatory cell infiltration, epithelial hyperplasia, crypt abscess, ulceration, and intestinal wall thickening [22, 23]. Compared with the traditional chemical reagents induction method, this model involves the interaction of genetic factors and immunity and can better simulate the multifactorial human IBD, which is of importance for exploring the pathogenesis of this disease [24]. In this study, piroxicam was added to the model feed at 200 ppm for 10 d to induce colitis in *IL-10*^−/−^ mice, and the mice in the model group showed significant weight loss, thin fecal matter, and positive fecal occult blood test [16]. H&E staining showed that the colonic epithelium of the model group showed obvious ulceration, disordered arrangement of glands, and a large amount of inflammatory cell infiltration, indicating the success of the model.

IEC has an abundant ER structure and is continuously stimulated by intestinal flora, mucosal inflammatory mediators, and other ER stressors [25]. Studies have shown that excessive ER stress in IECs may be involved in the pathogenesis of IBD [26]. Increased expression of ER stress markers has also been observed in colonic epithelial tissues of active IBD patients [27, 28]. Therefore, improving ER stress to restore intestinal homeostasis may be a potential therapeutic target for IBD. The PERK pathway mediated by ER stress plays a key role in the pathological mechanism of UC. When ER stress occurs, PERK is dissociated from GRP78, and the activated PERK phosphorylates eIF2*α*, which stops most protein synthesis in the cells, reduces the overall protein synthesis level, and increases the selectivity of ATF4 translation [11, 29]. *CHOP* gene is a transcription factor downstream of PERK and a direct target of ATF4, and studies have confirmed that initiation of CHOP is induced by the continuous activation of the PERK pathway [30]. When a severe UPR is caused, ATF4 upregulates *CHOP* gene expression and induces apoptosis. In this study, the phosphorylation of PERK and eIF2*α*, the expression of ATF4 and CHOP proteins, and the mRNA expression of PERK, eIF2*α*, ATF4, and Chop in the colon tissue of the model group were increased, suggesting that the PERK-eIF2*α*-ATF4-CHOP signaling pathway was activated. JPQCD could significantly downregulate the phosphorylation of PERK and eIF2*α*, the expression of ATF4 and CHOP proteins, and CHOP mRNA expression of PERK, eIF2*α*, ATF4, and CHOP so as to inhibit intestinal inflammation. Consistent results were obtained with immunofluorescence staining for the apoptotic gene CHOP, and JPQCD significantly reduced the fluorescent expression of CHOP in colonic sections. These results suggest that JPQCD alleviates ER stress and plays an anti-inflammatory role by inhibiting the PERK/eIF2*α*/ATF4/CHOP pathway.

The imbalance between proinflammatory and anti-inflammatory cytokines in IBD affects local intestinal inflammation and tissue damage. Blocking these inflammatory mediators is one of the mechanisms that reduce or even reverse the symptoms of IBD [31]. ER stress increases the production of cytokines, including IL-1*β*, IL-6, IL-8, and TNF-*α* [32, 33]. Studies have shown that CHOP can induce dendritic cells to secrete IL-23, which may promote the production of IL-17 by local T cells, thus triggering an innate immune response [34]. Under the condition of chronic inflammation, the expression of ER stress marker molecules GRP78 and p-eIF2*α* was increased in the intestinal epithelial cells of *IL-10*^−/−^ mice [35]. Therefore, we hypothesized that *IL-10*^*-/-*^ mice exposed to piroxicam produced a large number of inflammatory cytokines in the colon and damaged the intestinal mucosa. The barrier function of the intestinal mucosa is damaged, leading to excessive ER stress, causing intestinal inflammation, which in turn promotes cytokine secretion and further aggravates inflammation. IL-17 is a proinflammatory cytokine specifically secreted by helper T cells (Th17). When IL-17 specifically binds to IL-17R on the cell surface, it activates I*κ*B kinase, resulting in the phosphorylation of I*κ*B protein, ubiquitination and degradation, activation of NF-*κ*B signal transduction pathway, and mediation of inflammatory response [36]. Our results showed that the content of IL-17 and mRNA expression in colonic tissue of mice in the model group were significantly increased, confirming that IL-17 was involved in the piroxicam-induced experimental colitis in *IL-10*^−/−^ mice, and the intervention of JPQCD could reduce IL-17 level. Previous studies have shown that JPQCD can reduce DSS-induced acute colitis in mice by inhibiting the activation of NF-*κ*B [14]. In this study, the protein expression of NF-*κ*B, p- NF-*κ*B p65 and mRNA expression of NF-*κ*B in the colon tissues of *IL-10*^−/−^ induced by piroxicam were increased, while JPQCD significantly downregulated the expression of NF-*κ*B.

JPQCD is composed of nine TCMs, each of which contains a variety of compounds, including known anti-UC components, such as berberine, astragalus polysaccharide, and Codonopsis pilosula polysaccharide. Pharmacological studies showed that berberine, the active component of *Coptis chinensis*, could inhibit the Th17 response, protect the colon barrier function of mice, regulate the intestinal flora of mice, and have a significant protective effect on DSS-induced colon injury and inflammation in mice [37–39]. Astragalus polysaccharide can reduce the severity of DSS-induced colitis in mice, and this protective effect may be mediated by inhibiting the activation of NF-*κ*B [40]. *Codonopsis pilosula* polysaccharide and *Codonopsis pilosula* saponin, the main active components of *Codonopsis pilosula*, can synergistically regulate the balance of proinflammatory and anti-inflammatory cytokines, enhance the immune response of the body, and inhibit the colonization of pathogenic bacteria, thereby reducing the symptoms of colitis in UC mice [41]. Polysaccharide from *Scutellaria baicalensis* Georgi can improve colitis by inhibiting the NF-*κ*B signaling pathway and activation of NLRP3 inflammasome [42]. The synergistic effect of various compounds may be key to the role of JPQCD in the treatment of UC. In this study, JPQCD significantly improved UC symptoms, such as weight loss, increased DAI, and improved colonic shortening and colorectal bleeding in *IL-10*^−/−^ mice with piroxicam-induced colitis. In addition, JPQCD inhibited crypt epithelial deformation, goblet cell loss, inflammatory cell infiltration, and inflammatory response and alleviated histopathological lesions in the colonic mucosa and submucosa of mice. These results suggest that JPQCD is an ideal natural drug for the prevention and treatment of UC and other IBDs.

In this study, the efficacy of JPQCD did not increase with the dose. In most cases, a medium-dose JPQCD is more effective. Due to the complex components and effects of traditional Chinese medicine and the individual differences of animals and the limited number of samples, there is often no obvious dose-effect relationship in pharmacodynamics tests. The medium dose is the human equivalent dose, which is based on the experience of JPQCD and the experience of famous experts. This animal experiment has well verified the clinical efficacy of the drug.

## 5. Conclusion

In summary, IEC stress induced by piroxicam in *IL-10*^−/−^ mice is excessive and activates the PERK/eIF2*α*/ATF4/CHOP pathway. JPQCD can reduce ER stress, regulate the PERK/eIF2*α*/ATF4/CHOP pathway, repair the intestinal mucosal barrier in mice, and prevent and treat UC.

## Figures and Tables

**Table 1:** Disease activity index score.

Weight loss(%)	Stool consistency	Bleeding	Score
0	Normal pellets	Normal	0
1∼5	Loose feces	Occult blood positive	1
6∼10			2
11∼15	Watery diarrhea	Visible bleeding	3
>16			4

**Table 2:** List of primers used in this study.

Gene		Primer sequence
GRP78	Forward	TTGTCCCCTTACACTTGGTATTG
	Reverse	TGTCTTTTGTTAGGGGTCGTTC
PERK	Forward	GCTCAAAGACGAAAGCACAGAC
	Reverse	CCCACCGAGAAAGACCGAC
eIF2*α*	Forward	ACCTGGATACGGTGCCTACG
	Reverse	TCGAATTTTGACCGCTTGTG
ATF4	Forward	ATGGAGCAAAACAAGACAGCA
	Reverse	TGCCTTACGGACCTCTTCTATC
CHOP	Forward	AAACCTTCACTACTCTTGACCCTG
	Reverse	GGGCACTGACCACTCTGTTTC
*β*-actin	Forward	GAGACCTTCAACACCCCAGC
	Reverse	ATGTCACGCACGATTTCCC

**Table 3:** Identification results of main components of JPQCD.

NO	Retention time (min)	Adducts	Measured M/Z	Expected M/Z	ppm	Formula	Molecular weight	Phytochemical name	MS/MS spectra
1	1.81	[M-H]^−^	169.0158	169.0142	9.2	C_7_H_6_O_5_	170.02	Gallic acid	169.0168; 125.0250; 81.0368
2	5.29	[M-H]^−^	345.0832	345.0827	1.4	C_14_H_18_O_10_	346.09	Methyl 6-O-galloyl-*β*-D-glucoside	345.0814; 313.0536; 124.0170
3	9.15	[M-H]^−^	289.0737	289.0718	6.7	C_15_H_14_O_6_	290.08	Catechin	289.0721; 271.0594; 245.0833; 203.0725; 151.0401
4	11.26	[M-H]^−^	277.0034	277.0024	3.7	C_9_H_10_O_8_S	278.01	Gallic acid ethyl ester sulfate	277.0032; 197.0455; 182.0225; 166.9997; 123.0086
5	12.54	[M-H]^−^	289.0735	289.0718	6.0	C_15_H_14_O_6_	290.08	Epicatechin	289.0737; 245.0844; 203.0715; 151.0419
6	18.78	[M + FA-H]^−^	511.2434	511.2396	7.4	C_21_H_38_O_11_	466.24	Rhodioloside E	511.2700; 465.2368; 333.1949; 311.0997; 251.0743
7	20.40	[M-H]^−^	417.1220	417.1191	6.9	C_21_H_22_O_9_	418.13	Liquiritin	417.1190; 255.0670; 135.0101; 119.0510
8	20.41	[M + H]^+^	447.1256	447.1286	-6.7	C_22_H_22_O_10_	446.12	Calycosin-7-O-*β*-D-glucoside	285.0746; 270.0507
9	21.52	[M-H]^−^	619.2303	619.2244	9.6	C_27_H_40_O_16_	620.23	Dactylorhin E	619.2267; 439.1630; 171.0665; 153.0568
10	21.57	[M-H]^−^	549.1631	549.1614	3.2	C_26_H_30_O_13_	550.17	Liquiritin apioside	549.1600; 255.0650; 135.0084
11	22.16	[M + H]^+^	504.1466	504.15	-6.8	C_24_H_25_NO_11_	503.14	Oleracein A	342.0961; 147.0431; 85.0268
12	24.67	[M + H]^+^	534.159	534.1606	-3	C_25_H_27_NO_12_	533.15	Oleracein B	372.1090; 177.0529; 145.0260
13	27.62	[M-H]^−^	457.1737	457.1715	4.7	C_21_H_30_O_11_	458.18	Gymnoside II	457.1745; 285.0957; 189.0746; 171.0664; 153.0556
14	28.37	[M-H]^−^	457.1753	457.1715	8.2	C_21_H_30_O_11_	458.18	Gymnoside I	457.1737; 285.0976; 153.0576; 127.0770
15	28.55	M^+^	320.0897	320.0917	−6.4	C_19_H_14_NO_4_	320.09	Coptisine	320.0907; 292.0961; 277.0757; 249.0733
16	28.76	[M + FA-H]^−^	933.3300	933.3245	5.9	C_40_H_56_O_22_	888.33	Dactylorhin A	887.3237; 619.2214; 439.1595; 179.0563
17	29.04	M^+^	338.1364	338.1387	-6.8	C_20_H_20_NO_4_	338.14	Jatrorrhizine	338.1386; 322.1051; 208.0897; 294.1110; 279.0878
18	29.22	[M + FA-H]^−^	441.1797	441.1766	7.0	C_20_H_28_O_8_	396.18	Lobetyolin	305.1232; 215.1090; 185.0984; 159.0830; 143.0720
19	30.81	[M + FA-H]^−^	475.1273	475.1246	5.7	C_22_H_22_O_9_	430.13	Ononin	267.0671; 252.0399; 223.0389
20	30.96	[M-H]^−^	549.1657	549.1614	7.9	C_26_H_30_O_13_	550.17	Isoliquiritin apioside	549.1654; 255.0655; 135.0104
21	31.72	[M + FA-H]^−^	977.5402	977.5327	7.7	C_47_H_80_O_18_	932.53	Notoginsenoside R1	977.5391; 931.5254; 799.4847; 637.4346
22	32.16	[M + H]^+^	314.1388	314.1387	0.4	C_18_H_19_NO_4_	313.13	N-trans-Feruloyltyramine	314.1417; 177.0538; 145.0276; 121.0637
23	32.34	M^+^	352.1528	352.1543	-4.4	C_21_H_22_NO_4_	352.15	Palmatine	352.1538; 336.1192; 322.1032; 308.1216; 294.1076
24	32.44	M^+^	336.1216	336.1230	-4.3	C_20_H_18_NO_4_	336.13	Berberine	336.1213; 320.0906; 292.0947; 276.1035
25	32.69	[M + FA-H]^−^	771.2747	771.2717	3.9	C_34_H_46_O_17_	726.27	Militarine	725.2715; 457.1722; 285.0982; 153.0566
26	32.83	[M + FA-H]^−^	845.4934	845.4904	3.5	C_42_H_72_O_14_	800.49	Ginsenoside Rg1	845.4965; 799.4888; 637.4343; 475.3814
27	33.16	[M-H]^−^	408.9890	408.9871	4.6	C_16_H_10_O_11_S	409.99	3,3′-Di-O-methylellagic acid sulfate	329.0298; 314.0055; 298.9820; 286.0127
28	34.34	[M + FA-H]^−^	493.2308	493.2291	3.5	C_21_H_36_O_10_	448.23	Geraniol 1-O-*α-*L-arabinofuranosyl-(1⟶6)-*β*-D-glucopyranoside	493.2369; 447.2270; 315.1806; 221.0679; 179.0579
29	35.30	[M-H]^−^	423.0051	423.0028	5.5	C_17_H_12_O_11_S	424.01	2,3,8-Tri-O-methylellagic acid sulfate	423.0065; 343.0475; 328.0216; 312.9979; 297.9731
30	38.58	[M-H]^−^	983.4580	983.4493	8.8	C_48_H_72_O_21_	984.46	Licorice saponin A3	983.4449; 821.3791
31	39.35	[M + FA-H]^−^	815.4855	815.4798	6.9	C_41_H_70_O_13_	770.48	Ginsenoside F3	815.4920; 769.4752; 637.4285; 475.3756
32	39.79	[M-H]^−^	837.3954	837.3914	4.7	C_42_H_62_O_17_	838.40	Licoricesaponin G2	837.3894; 351.0538; 193.0391
33	40.40	[M + FA-H]^−^	683.4427	683.4376	7.5	C_36_H_62_O_9_	638.44	Ginsenoside Rh1	683.4435; 637.4295; 475.3811
34	40.50	[M + FA-H]^−^	1153.6127	1153.6011	10.0	C_54_H_92_O_23_	1108.60	Ginsenoside Rb1	1153.6143; 1107.5910; 945.5482
35	40.62	[M + FA-H]^−^	811.4566	811.4485	9.9	C_41_H_66_O_13_	766.45	Ziyuglycoside I	811.4522; 765.4467; 603.3907; 207.0512
36	40.88	[M + FA-H]^−^	683.4404	683.4376	4.1	C_36_H_62_O_9_	638.44	Ginsenoside F1	683.4427; 637.4368; 475.3848
37	41.46	[M-H]^−^	837.3983	837.3914	8.2	C_42_H_62_O_17_	838.40	Licoricesaponin Q2	837.3965; 351.0573
38	41.99	[M + FA-H]^−^	829.4671	829.4591	9.6	C_41_H_68_O_14_	784.46	Astragaloside A	829.4714; 783.4561; 489.3715
39	42.95	[M + FA-H]^−^	991.5574	991.5483	9.2	C_48_H_82_O_18_	946.55	Ginsenoside Rd	991.5596; 945.5476; 783.5021; 621.4569
40	43.09	[M-H]^−^	821.4006	821.3965	5.0	C_42_H_62_O_16_	822.40	Glycyrrhizic acid	821.3926; 351.0580
41	44.57	[M-H]^−^	821.4037	821.3965	8.8	C_42_H_62_O_16_	822.40	Uralsaponin B	821.3966; 351.0582
42	49.14	[M + FA-H]^−^	829.5022	829.4955	8.1	C_42_H_72_O_13_	784.50	Ginsenoside Rg3	829.5000; 783.4923; 621.4401
43	50.43	[M + H] ^+^	231.1365	231.138	-6.3	C_15_H_18_O_2_	232.15	Dehydrocostus Lactone	231.1381; 185.1323; 165.0682; 128.0604; 105.0691
44	50.78	[M-H]^−^	231.1411	231.1391	8.9	C_15_H_20_O_2_	232.15	Costunolide	231.1407; 213.1291

## Data Availability

The datasets generated during and/or analyzed during the current study will be available upon request from the principle investigator. The shared data will only be allowed to be used by the applicant for scientific studies. No commercial activities are allowed.

## Abbreviations

ER: Endoplasmic reticulum
IEC: Intestinal epithelial cells
UC: Ulcerative colitis
JPQCD: Jianpi Qingchang decoction
RT-PCR: Reverse transcription-polymerase chain reaction
ELISA: Enzyme-linked immunosorbent assay
IBD: Inflammatory bowel disease
TCM: Traditional Chinese medicine
UPR: Unfolded protein response
PERK: Protein kinase-related endoplasmic reticulum kinase
IRE1: Inositol-requiring enzyme 1
ATF6: Activating transcription factor 6
GRP78: Glucose-regulated protein 78
DSS: Dextran sodium sulfate
NF-*κ*B: Nuclear factor-*κ*B
IL-1*β*: Interleukin-1*β*
DAI: Disease activity index
H&E: Hematoxylin and eosin
NSAIDS: Nonsteroidal anti-inflammatory drugs.
